# Specialized Treatment applied for suicide prevention

**DOI:** 10.1192/j.eurpsy.2022.2178

**Published:** 2022-09-01

**Authors:** J. Jaber

**Affiliations:** Clínica Jorge Jaber, Saúde Mental, Rio de Janeiro, Brazil

## Abstract

**Introduction:**

Suicide can be defined as a deliberate act performed by the individual, whose intention is the death, in a conscious, intentional, even if ambivalent way, using a means that he believes to be lethal. They are also part of what we usually call suicidal behavior: thoughts, plans and attempted suicide.

**Objectives:**

Prevention is a critical step in treating suicidal behavior. Create strategies to reduce and treat the ideation, planning and suicide attempt.

**Methods:**

Based on a large increase in the number of people who present ideas, plan and attempt suicide, the Clinic created techniques for the treatment of inpatients: Life Appreciation Group, Groups applying Cognitive Behavioral Therapy, Group Dynamics, Lectures, Art Therapy and Physical activities .

**Results:**

The actions are developed by a multidisciplinary team that is divided by applying the various techniques and participating in all the proposed activities.

**Conclusions:**

Patients who remained hospitalized fully complying with the suggested treatment and left with medical discharge had full benefit, unlike some cases of patients removed by the family against our indication.

**Disclosure:**

No significant relationships.

